# Association of Polymorphisms of Metabolism-Related Genes with Psoriasis Vulgaris in Han Chinese

**DOI:** 10.1155/2021/9920631

**Published:** 2021-09-18

**Authors:** Yu Liu, Xin Li, Yan-Ping Huang, Zi-Yu Cui, Jia Bao, Xue-Li Zhang, Yan Guo, Min-Jing Su, Xin-Xiang Lv, Jian-Wen Han

**Affiliations:** Department of Dermatology, Affiliated Hospital of Inner Mongolia Medical University, Hohhot, Inner Mongolia, China

## Abstract

**Aim:**

Psoriasis is a chronic inflammatory disease with a complex etiology, and psoriasis vulgaris (PsV) is the most common type of psoriasis. Recent studies suggest the relationship between psoriasis and metabolic syndrome in different ethnicities. This study is aimed at evaluating the association of metabolism-related gene variants with the risk of PsV in Chinese Han population. *Material and Methods*. PsV patients (1030) and healthy controls (965) were enrolled in this study. Eighteen single-nucleotide polymorphisms (SNPs) previously reported to be significantly associated with metabolic syndrome were selected. SNPs were detected by next-generation sequencing.

**Results:**

Seven SNPs were significantly associated with PsV: rs805303 (*P* = 0.012, OR = 0.85), rs3177928 (*P* = 1.37 × 10^−15^, OR = 2.51), and rs2247056 (*P* = 3.73 × 10^−4^, OR = 0.67) located in the HLA gene region; rs1047781 (*P* = 0.012, OR = 1.18), rs281379 (*P* = 0.014, OR = 1.71), and rs492602 (*P* = 0.005, OR = 1.86) located in the FUT2 region; and rs2303138 (*P* = 0.014, OR = 1.18) located in the LNPEP region. After stratified analysis, rs805303 (*P* = 0.017, OR = 0.74) and rs2303138 (*P* = 0.041, OR = 1.30) were associated with PsVs when HLA-C^∗^06 : 02 was positive, and rs805303 (*P* = 5.62 × 10^−5^, OR = 0.68), rs3177928 (*P* = 0.003, OR = 1.75), rs281379 (*P* = 0.034, OR = 1.96), and rs492602 (*P* = 0.025, OR = 2.04) were associated with PsVs when HLA-C^∗^06 : 02 was negative.

**Conclusion:**

PsV and metabolic syndrome may have overlapped susceptible genes in Chinese Han population.

## 1. Introduction

Psoriasis is a chronic inflammatory disease caused by various factors including genetic factors, with psoriasis vulgaris (PsV) representing the most common type [1]. Recently, it is reported that psoriasis is related to metabolic syndrome. The association of metabolic syndromes such as dyslipidaemia, hypertension, insulin resistance, and abdominal obesity with psoriasis has been investigated. Lu et al. analyzed the top 10 (the top three were in the HLA region) metabolic single-nucleotide polymorphisms (SNPs) associated with psoriasis by genome-wide association study [2]. A meta-analysis of the association of angiotensin-converting enzyme (ACE) gene polymorphisms with psoriasis susceptibility showed that the polymorphisms were associated with the risk of psoriasis in Asians [3]. Angiotensin II type 1 receptor (AT1R) is correlated with hypertension, heart disease, and oxidative stress, and Mohammadi et al. reported that AT1RA1166C (rs5186) polymorphism significantly increased the risk of psoriasis [4]. Cheng et al. found that rs2303138 polymorphism in the LNPEP gene was associated with renin angiotensin and played a key role in cardiovascular disease and diabetes mellitus [5].

However, due to ethnic differences, previous studies reported different results on the association of SNPs with PsV. In particular, very few studies examined the association of metabolic disease-related SNPs with PsV in the Chinese Han population. Therefore, this study is aimed at evaluating the association between metabolic disease-related SNPs and the risk of PsV in the Chinese Han population. We selected 18 SNPs previously reported to be significantly associated with metabolic diseases. In the HLA region, HLA-cw06 : 02 is the major psoriasis risk gene, which has been validated in different populations worldwide [6]. Therefore, we performed a stratified analysis to evaluate the interaction between HLA-cw06 : 02 and the selected SNPs.

## 2. Material and Methods

### 2.1. Subjects

The study population consisted of 1030 patients with PsV and 965 healthy controls, who visited Affiliated Hospital of Inner Mongolia Medical University from Dec 2011 to May 2018. All subjects recruited were all Chinese Han more than 18 years old, both men and women. PsV patients were diagnosed by at least two clinical dermatologists based on histopathology. Healthy controls visited the hospital for physical examination. All subjects were excluded if they had familial hypertension, primary diabetes type I, or familial hyperlipidemia. The study was approved by the Ethical Committee of Affiliated Hospital of Inner Mongolia Medical University and conducted according to the Declaration of Helsinki principles. All subjects signed informed consent.

### 2.2. Second-Generation Sequencing

Peripheral venous blood (4 ml) samples were collected from all subjects and stored at-80°C until use. Genomic DNA was extracted from blood samples by using whole-blood DNA extraction kit (AxyPrep, AP-MX-BL-GDNA-25, Suzhou, China). The information of all the SNPs was obtained from PubMed database (https://www.ncbi.nlm.nih.gov/pubmed); then, the primers and probes were designed using Primer3 online (version 0.4.0; http://frodo.wi.mit.edu). The primer sequences for were listed in Table 1.

PCR reaction system consisted of the follows: 1 *μ*l of DNA template, 2 *μ*l of buffer, 0.6 *μ*l Mg^2+^, 2 *μ*l dNTP, 0.2 *μ*l Taq DNA polymerase, 2 *μ*l primer solution, and 12.2 *μ*l water. PCR was performed by initial denaturation at 95°C for 2 min, denaturation at 94°C for 30 s, annealing at 59°C for 90s, extension at 72°C for 60 s for 40 cycles, and followed by final extension at 72°C for 10 min. Amplified products were sequenced by using the genome analyzer (PRISM3730 ABI), and sequencing results were analyzed by using Ion Torrent PGM platform.

### 2.3. Statistical Analysis

Statistical analyses were performed using the PLINK1.07 package (http://pngu. mgh. harvard. Edu/purcell/plink/, USA). The frequencies of the alleles of all 19 SNPs were calculated, and the Hardy-Weinberg equilibrium was tested in the cases and controls. Minor allele frequencies (MAF) of each SNP were compared between patients and controls using the Chi-square test. Odds ratio (OR) and the corresponding 95% confidence intervals (95% CI) were calculated. Bonferroni corrected *P* value threshold of *P* < 0.05 was set based on multiple tests of 19 genotyped variations.

## 3. Results

A total of 1030 patients with PsV (532 males and 498 females, mean age: 45.49 ± 17.11 years) and 965 controls (542 males and 423 females, mean age: 45.86 ± 11.59 years) were recruited in this study. There were no significant differences in age and gender between the two groups (*P* > 0.05). The minor allele frequencies of all 18 SNPs were compared between the two groups. Seven SNPs showed significant difference between the two groups: rs805303 (*P* = 0.012, OR = 0.85), rs3177928 (*P* = 1.37 × 10^−15^, OR = 2.51), and rs2247056 (*P* = 3.73 × 10^−4^, OR = 0.67) in HLA; rs1047781 (*P* = 0.012, OR = 1.18), rs281379 (*P* = 0.014, OR = 1.71), and rs492602 (*P* = 5.32 × 10^−3^, OR = 1.86) in FUT2; and rs2303138 (*P* = 0.014, OR = 1.18) in LNPEP. The other 11 SNPs showed no significant difference between the two groups (*P* > 0.05). The frequencies of rs3177928 A allele, rs1047781 T allele, rs281379 A allele, rs492602 C allele, and rs2303138 A allele were significantly higher in cases than in controls (*P* < 0.05). However, the frequencies of rs805303 T allele and rs2247056 T allele were significantly lower in cases than in controls (*P* < 0.05) (Table 2).

The strongest associations of SNPs and PsV were identified as *HLA*-C^∗^06 : 02 (*P* = 7.82 × 10^−170^, OR = 7.80), rs3177928 (*P* = 1.37 × 10^−15^, OR = 2.51), and rs2247056 (*P* = 3.73 × 10^−4^, OR = 0.67), which were all located in the HLA region. In order to identify whether the result was interfered by HLA-cw06 : 02, we performed stratified analysis. All the subjects were stratified into HLA positive and HLA negative, and there were 694 PsV patients and 175 controls with HLA-cw06 : 02 (+), 336 PsV patients, and 790 controls with HLA-cw06 : 02 (-). The results showed that in the positive group rs805303 (*P* = 0.017, OR = 0.74) and rs2303138 (*P* = 0.041, OR = 1.299) were associated with PsV, while in the negative group rs805303 (*P* = 5.62 × 10^−5^, OR = 0.68), rs3177928 (*P* = 0.003, OR = 1.75), rs281379 (*P* = 0.034, OR = 1.95), and rs492602 (*P* = 0.025, OR = 2.04) were related to PsV (*P* < 0.05) (Table 3).

## 4. Discussion

SNPs have been involved in a variety of diseases including PsV [1, 7, 8]. In this study, we investigated 18 polymorphisms reported in previous studies to be associated with metabolic syndrome and examined them in a Chinese Han population. Seven SNPs were found to be significantly associated with PsV patients, including rs805303, rs3177928, and rs2247056 in HLA loci; rs1047781, rs281379, and rs492602 in FUT2 loci; and rs2303138 in LNPEP loci.

rs492602 in FUT2 loci showed significant association with PsV susceptibility, in agreement with the report by Lu et al. [2]. The FUT2 gene is located at 19q13.33, and it encodes alpha-(1,2)-fucosyltransferase which regulates H antigen (the precursor of the human ABO blood group antigens) on the surface of epithelial cells [9]. FUT2 has relationship with dyslipidemia due to the regulation of glycosphingolipid synthesis. Furthermore, GWAS analysis showed genetic overlap between dyslipidemia and a series of archetypal immune-mediated diseases such as Crohn's disease, ulcerative colitis, rheumatoid arthritis, type 1 diabetes, celiac disease, psoriasis, and sarcoidosis [10]. FUT2 gene polymorphism may be involved in metabolic syndrome and psoriasis through the regulation of dyslipidemia. Interestingly, all three FUT2 SNPs were associated with psoriasis only in the absence of HLA-C^∗^06 : 02. We speculate that the role of HLA-C in antigen presentation may interfere with the regulation of H antigen by FUT2, but further studies are needed to confirm this.

We found that the A allele of rs2303138 in LNPEP was associated with PsV, coincident with the results by Cheng et al. [5]. LNPEP (leucyl and cystinyl aminopeptidase) could promote glucose uptake through insulin-stimulated interaction with glucose transporter GLUT4. In addition, LNPEP is involved in MHC class I cross-presentation of exogenous antigens [11]. Interestingly, the fact that rs2303138 was associated with psoriasis only in the presence of HLA-C^∗^06 : 02, but not in its absence, suggests a role in immune reaction such as antigen cross-presentation, rather than in the metabolism of angiotensin and glucose.

LNPEP is an angiotensin IV receptor, which is an important component of the renin-angiotensin system (RAAS) [12]. RAAS regulates blood pressure, electrolyte, and fluid homeostasis [13]. Another RAAS-related gene is ACE, and the human ACE gene is located on chromosome17q23 and comprises 26 exons and 25 introns [14]. The most common polymorphism in the ACE gene is the insertion/deletion (I/D, rs4646994) polymorphism located on intron 16 [15]. Raza et al. found that rs4646994 polymorphism in the ACE gene was associated with dyslipidemia in type 2 diabetes mellitus (T2DM) patients [16]. However, rs4646994 was not associated with PsV in our present study. The different results might be due to the differences in disease, race, and region.

Three SNPs rs805303, rs3177928, and rs2247056 located in the HLA region showed association with PsV. Numerous studies have shown that HLA-Cw^∗^06 : 02 is the major allele susceptible to psoriasis in different populations [17]. To clarify whether the association between the SNPs and PsV is due to the interaction with HLA-Cw^∗^06 : 02, we divided the subjects into HLA-Cw^∗^06 : 02-positive and HLA-Cw^∗^06 : 02-negative groups and conducted stratified analysis. The results showed that rs805303 and rs2303138 were related to PsV when HLA-Cw^∗^06 : 02 was positive, while rs805303, rs118179173, and rs3177928 were associated with PsV when HLA-Cw^∗^06 : 02 was negative. It remains unclear why HLA region SNP rs805303 was associated with psoriasis independently of the presence of HLA-C^∗^06 : 02, in contrast to rs3177928, also from the HLA region, which was associated with psoriasis only in the absence of HLA-C^∗^06 : 02 negatives. Further functional studies are needed to explain the results. Furthermore, rs2247056 was also in the HLA region and it was associated with psoriasis before stratification but lost this association after stratification. Our results are consistent with previous report by Lu et al. who showed that after adjustment for the top psoriasis risk allele HLA-C^∗^06 : 02, the associations for rs2247056 was mitigated while the association for rs805303 persisted [2]. Because multiple alleles associated with psoriasis risk are identified in HLA loci, it is possible that significant association of rs805303 with psoriasis is due to linkage disequilibrium with other HLA alleles associated with psoriasis risk [18].

In conclusion, we examined 18 previously reported SNPs of metabolic syndrome in a Chinese Han population and found that 7 SNPs located in HLA, FUT2, and LNPEP were associated with PsV in Chinese Han.

## Figures and Tables

**Table 1:** PCR primer sequences.

CHR	SNP	Gene	Forward primer	Reserve primer
2	rs7593730	*RBMS1*	ATCTGTTGTCCATGCTAACACG	TGTTGACAATAGCAAAATTGAAGG
3	rs5186	*AT1R*	GAGCAAGAGAACATTCCTCTGC	AGCCGTCATCTGTCTAATGCAA
5	rs2303138	*LNPEP*	TTAGCCTAGGCAAGGTACCTCTC	CTTGAGGCCAGATCCATGTATC
6	rs805303	*HLA*	GCAAGGCTGGCTAGGGTCT	GGCAGAAATGAGAGAGCCTCAC
6	rs3177928	*HLA*	GAAGAGGAAGGAATCTGAAGCA	TGAGGTCAAGGATTGTAATATTGC
6	rs6931514	*CDKAL1*	CTATAATTTTAGTATGTCAATGACTACAGC	TACACTTACTACAGTCTCCTCTCCAAA
6	rs2247056	*HLA*	ATGGCCAGCCATCAGGG	TTGTCTCAAAGTCTCCAAATTGTAC
10	rs4506565	*TCF7L2*	AATAAGCAAAGAAGTGAATGTTGTG	AAATATTGAGGCTCTCCTCGG
10	rs7901695	*TCF7L2*	CAGTCACTCCTCATTTCCTCTCC	AAAGGAATGACATACTGATAGATGCT
12	rs3184504	*SH2B3*	AGCCTTGAGTACCCCAACTTG	CCCCAGGGTGTGAAAAGC
12	rs653178	*ATXN2, SH2B3*	TTAAATATTTCAGTTCCTCAGCCA	CAACTCAGGTAGAAAAGGATCTTAGG
12	rs11065987	*BRAP*	CCAAGTCCCAAAGTTAGGGGA	AATTCCAAGTGATCCTCCCAC
16	rs255049	*LCAT*	TCCAAGGCCCCACTCTCC	GGGTGCCCAGGTACCACAG
19	rs1047781	*FUT2*	CCACGGCCAGCAGGATC	ATCTCCTGGCGGAGGTGGT
19	rs281379	*FUT2*	TCTTCCCAGGACTGCCCTT	GGGCACTATACAAATCTGTGAATATC
19	rs492602	*FUT2*	CCGTTCATCTTGGCCAGG	ACCGGTGCAGATACCAGTGC
22	rs181362	*UBE2L3*	TGGGATAAGGGCTGTCAAAAG	GATTCGGTGGCTGTTTGGG

**Table 2:** Comparison of SNPs and *HLA*-C^∗^06:02 in PsV cases and controls.

CHR	Gene	SNP	Allele	MAF		*χ* ^2^	*P*	OR	95% CI
				Case	Control				
2	*RBMS1*	rs7593730	T	0.186	0.184	0.02	0.886	1.01	0.86-1.20
3	*AT1R*	rs5186	C	0.057	0.061	0.28	0.594	0.93	0.71-1.22
5	*LNPEP*	rs2303138	A	0.449	0.409	6.04	0.014^∗^	1.18	1.03-1.34
6	*HLA*	rs2247056	T	0.078	0.112	12.66	3.73*E* − 04^∗^	0.67	0.53-0.83
6	*HLA*	rs3177928	A	0.137	0.060	63.81	1.37*E* − 15^∗^	2.51	1.99-3.17
6	*CDKAL1*	rs6931514	G	0.505	0.494	0.39	0.533	1.04	0.92-1.19
6	*HLA*	rs805303	T	0.398	0.439	6.27	0.012^∗^	0.85	0.74-0.96
10	*TCF7L2*	rs4506565	T	0.037	0.041	0.57	0.451	0.88	0.63-1.23
10	*TCF7L2*	rs7901695	C	0.037	0.040	0.28	0.598	0.91	0.65-1.28
12	*BRAP*	rs11065987	G	0.012	0.008	1.71	0.192	1.55	0.80-3.02
12	*SH2B3*	rs3184504	T	0.014	0.009	1.87	0.171	1.54	0.83-2.88
12	*ATXN2, SH2B3*	rs653178	G	0.014	0.008	2.46	0.117	1.65	0.88-3.12
16	*LCAT*	rs255049	C	0.146	0.142	0.10	0.757	1.03	0.86-1.24
17	*ACE*	rs4646994	Delete	0.263	0.269	0.14	0.712	0.97	0.83-1.14
19	*FUT2*	rs1047781	T	0.489	0.447	6.36	0.012^∗^	1.18	1.04-1.35
19	*FUT2*	rs281379	A	0.030	0.018	6.01	0.014^∗^	1.71	1.11-2.65
19	*FUT2*	rs492602	C	0.030	0.017	7.80	0.005^∗^	1.86	1.20-2.89
22	*UBE2L3*	rs181362	A	0.425	0.422	0.04	0.850	1.01	0.89-1.16
6	*HLA-C* ^∗^ *06:02*	/	+	0.620	0.173	771.70	7.82*E* − 170^∗^	7.80	0.08-6.70

Notes: ^∗^*P* < 0.05.

**Table 3:** Comparison of 18 SNPs between PsV cases and controls stratified for HLA-cw06 : 02(+) and HLA-cw06 : 02(-).

CHR	SNP	Gene	*HLA*-C^∗^06:02(+)							*HLA*-C^∗^06:02(-)						
			MAF(+)		*χ* ^2^	*P*	OR	95% CI		MAF(-)		*χ* ^2^	*P*	OR	95% CI	
			PsV	Con						PsV	Con					
2	rs7593730	*RBMS1*	0.196	0.182	0.29	0.589	1.09	0.79-	1.50	0.171	0.185	0.58	0.445	0.91	0.72-	1.16
3	rs5186	*AT1R*	0.056	0.070	0.87	0.350	0.79	0.48-	1.30	0.058	0.060	0.03	0.861	0.97	0.66-	1.42
5	rs2303138	*LNPEP*	0.452	0.388	4.18	0.041	1.30	1.01-	1.67	0.448	0.414	2.22	0.136	1.15	0.96-	1.38
6	rs2247056	*HLA*	0.064	0.049	1.06	0.304	1.34	0.77-	2.34	0.099	0.126	3.25	0.072	0.76	0.57-	1.03
6	rs3177928	*HLA*	0.176	0.136	2.80	0.094	1.35	0.95-	1.92	0.075	0.044	8.83	0.003	1.75	1.21-	2.55
6	rs6931514	*CDKAL1*	0.493	0.515	0.52	0.472	0.91	0.71-	1.17	0.496	0.497	0.01	0.932	0.99	0.83-	1.19
6	rs805303	*HLA*	0.441	0.515	5.68	0.017	0.74	0.58-	0.95	0.332	0.423	16.23	5.62E-05	0.68	0.56-	0.82
10	rs4506565	*TCF7L2*	0.036	0.033	0.04	0.841	1.07	0.54-	2.12	0.039	0.044	0.28	0.600	0.88	0.56-	1.40
10	rs7901695	*TCF7L2*	0.036	0.030	0.22	0.641	1.18	0.58-	2.40	0.039	0.043	0.15	0.698	0.91	0.57-	1.45
12	rs11065987	*BRAP*	0.014	0.009	0.47	0.495	1.54	0.44-	5.35	0.009	0.008	0.14	0.710	1.21	0.45-	3.23
12	rs3184504	*SH2B3*	0.016	0.009	0.95	0.331	1.82	0.53-	6.23	0.009	0.009	0.00	0.988	1.01	0.39-	2.63
12	rs653178	*ATXN2, SH2B3*	0.017	0.009	0.98	0.323	1.84	0.54-	6.29	0.009	0.008	0.04	0.851	1.10	0.42-	2.90
16	rs255049	*LCAT*	0.147	0.146	0.01	0.939	1.01	0.72-	1.44	0.144	0.140	0.07	0.798	1.03	0.80-	1.34
17	rs4646994	*ACE*	0.259	0.266	0.05	0.816	0.97	0.72-	1.30	0.271	0.269	0.01	0.914	1.01	0.82-	1.26
19	rs1047781	*FUT2*	0.490	0.455	1.26	0.261	1.15	0.90-	1.48	0.490	0.444	3.85	0.050	1.20	1.00-	1.44
19	rs281379	*FUT2*	0.031	0.036	0.23	0.634	0.85	0.44-	1.66	0.027	0.014	4.50	0.034	1.95	1.04-	3.67
19	rs492602	*FUT2*	0.032	0.033	0.01	0.904	0.96	0.48-	1.91	0.027	0.013	5.05	0.025	2.04	1.08-	3.86
22	rs181362	*UBE2L3*	0.418	0.364	3.01	0.083	1.26	0.97-	1.62	0.437	0.434	0.02	0.889	1.01	0.84-	1.22

## Data Availability

All data are available upon request.
